# Comparative effect of high intensity interval training and moderate intensity continuous training on metabolic improvements and regulation of Cidea and Cidec in obese C57BL/6 mice

**DOI:** 10.1371/journal.pone.0322634

**Published:** 2025-04-30

**Authors:** Xi Lu, Yonglian Chen, Qingxing Xie, Nanwei Tong

**Affiliations:** Department of Endocrinology and Metabolism, Laboratory of Diabetes and Metabolism Research, West China Hospital, Sichuan University, Chengdu, China; Universidade do Estado do Rio de Janeiro, BRAZIL

## Abstract

Obesity is a chronic disease associated with increased risk of cardiovascular disease, diabetes, metabolic dysfunction associated steatotic liver disease and certain cancers. High intensity interval training (HIIT) and moderate intensity continuous training (MICT) are effective in preventing and managing obesity. However, the comparative effects of these modalities on metabolic disorders need to be better mechanistically explored. This study aimed to comprehensively assess the effects of MICT and HIIT on key metabolic organs and underlying mechanisms. C57BL/6 mice were randomized to receive either a chow diet or high fat diet for 12 weeks, followed by random assignment of high-fat-fed mice to no exercise, MICT or HIIT groups for additional 5 weeks. At the end, both HIIT and MICT significantly alleviated high-fat-induced weight gain and lipids disorder and impaired liver function. HIIT was more effective in enhancing insulin sensitivity, ameliorating hepatic steatosis, reducing adipocyte hypertrophy. Additionally, HIIT restored the high-fat-induced downregulation of Cidea, Cidec and Atgl in inguinal white adipose tissue and liver. Furthermore, HIIT resulted in upregulation of interleukin 6 (Il-6) in skeletal muscle. The exogenous addition of Il-6 to primary white adipocytes significantly downregulated Cidec, and up-regulated Atgl expression. In conclusion, HIIT is superior to MICT in improving metabolic dysfunction, likely mediated through Il-6-induced downregulation of Cidea and Cidec, thereby promoting lipolysis.

## Introduction

Obesity affects approximately 800 million individuals worldwide and has emerged as a significant global health burden, with its prevalence continuing to rise [1]. By 2025, it is anticipated that 18% of men and 21% of women worldwide will have obesity [1]. Obesity is associated with numerous chronic diseases, including cardiovascular disease, diabetes, metabolic dysfunction associated steatotic liver disease (MASLD) and certain cancers [2,3]. Exercise has been well-established as an effective intervention for weight loss and subsequent metabolic improvement [1]. However, the effects of exercise are influenced by various factors such as intensity, frequency, and duration, and no agreement has been reached regarding the most effective approach for address obesity.

Moderate intensity continuous training (MICT) and high intensity interval training (HIIT) have garnered increased attention in recent years as two prominent forms of exercise. MICT is characterized by continuous bouts of moderate-intensity aerobic activity at a steady state of set duration [4]. As a traditional form of exercise, MICT has been the subject of numerous studies demonstrating its benefits to cardiorespiratory fitness. And HIIT, defined by multiple repetitions of brief bouts of high-intensity exercise (80%-100% of peak heart rate, interspersed with rest) [4,5] has also been shown to yield comparable or even superior effects on cardiorespiratory fitness while requiring less time commitment [6,7]. Consequently, HIIT has gained popularity recent years due to its efficiency and less time-consuming characteristics. Previous studies have investigated the effects of MICT and HIIT on body composition, lipids profiles, and insulin sensitivity; however, these findings have not been entirely consistent. There remains considerable debate regarding the effectiveness of MICT versus HIIT for weight and fat loss [8–12], and the effects on lipid profiles also remain inconsistent [13–15]. Both MICT and HIIT have demonstrated reductions in insulin resistance, yet the comparative efficacy of the two was inconclusive [15–18]. Additionally, the specific mechanisms by which MICT and HIIT improve metabolic dysfunction remain unclear.

Previous studies have shown that exercise modulates lipid droplets (LD) dynamics, which plays a critical role in systemic metabolic regulation [19,20]. Cell death-inducing DFF45-like effectors (Cide) family, consisting of three structurally homologous members Cidea, Cideb, and Cidec, are localized on LD surfaces and promote LD fusion through the formation of homo- and heterodimeric channels in a lipid exchange manner [21–24]. Cidea and Cidec are predominantly expressed in brown adipose tissue (BAT) and white adipose tissue (WAT), respectively [25], and are also highly expressed in the liver during hepatic steatosis [26]. The knockout of Cidea and Cidec respectively in mice has been shown to result in a lean phenotype and resistance to high-fat diet (HFD)-induced obesity, accompanied by increased insulin sensitivity [23,27]. Conversely, Cidec knockout mice have also been demonstrated to exhibit decreased lipid storage capacity, accompanied by dyslipidemia and insulin resistance [28,29]. Despite these bidirectional results, the studies indicate that Cidea and Cidec play a physiological role in lipid storage.

While genetic ablation of Cidea or Cidec in mice alters adiposity and insulin sensitivity, their responses to exercise modalities remain unexplored. A prior study reported that exercise reduce the expression of Cidea and Cidec in the liver and alleviate MASLD by regulating LD [30]. However, it is unknown whether HIIT and MICT differentially regulate Cidea/Cidec expression across metabolic organs (e.g., adipose tissue, liver, skeletal muscle), and the underlying mechanisms involved. Therefore, it is essential to conduct a comprehensive investigation into the effects of MICT and HIIT on the body’s key metabolic organs (adipose tissue, liver and skeletal muscle) and explore underlying mechanisms on Cidea and Cidec.

## Materials and methods

### Animals

20 male C57BL/6 mice (4 weeks old) were purchased and housed in the specific-pathogen-free environment. At the age of 5 weeks, mice were randomly assigned into high-fat diet (HFD) group (n = 15, 60% fat, 20% protein, 20% carbohydrate) or chow diet (CD) group (n = 5, 10% fat, 20% protein, 70% carbohydrate) (XTHF60 vs XTCON50J, Xietong Shengwu, Jiangsu, China). The full nutritional fact sheet are provided in S1 Table in S1 File. 12 weeks later, mice in the HFD group will be randomly assigned into three groups (n = 5) for MICT or HIIT or no exercise training for 5 weeks. Therefore, there are four groups including CD, HFD, HFD+MICT (short for MICT), HFD+HIIT in this experiment. Animals were euthanized after receiving isoflurane anesthesia and blood sampling, and the target organs were taken and preserved appropriately. All animal experiments were carried out in strict accordance with guidelines of laboratory animal welfare and ethics. The protocol was approved by Experimental Animal Ethics Committee of West China Hospital of Sichuan University (Protocol Number: 20241220002).

### Exercise training

One week before the initiation of formal exercise, mice in the exercise group were acclimated to a static treadmill for 10 minutes daily over 2 days. This was followed by 3 days of adaptive training, at 5 m/min for 10 minutes on the first day; at 5 m/min for 5 minutes, 10 m/min for 5 minutes, and 5 m/min for 5 minutes on the second day; at 5 m/min for 5 minutes, 15 m/min for 5 minutes, and 5 m/min for 5 minutes on the third day. Maximal running capacity test was performed after the adaptive training [31]: a warm-up phase of 5 m/min for 5 minutes, followed by an even speed increase at 2 m/min every 2 minutes until the mice became exhausted and no longer wished to be active (mice no longer ran for more than 5 seconds after two consecutive gentle tapping), the maximum speed (Vmax) was recorded at this point. The average of Vmax of mice in the exercise groups is 21 m/min. During formal exercise, HIIT group trained in 7 cycles, beginning with 2 minutes at 5 m/min, followed by 2 minutes at 18 m/min (85% Vmax). The MICT group started with 5 minutes at 5 m/min, followed by 30 minutes at 9 m/min (45% Vmax), and concluded with 5 minutes at 5 m/min. The mice were encouraged to run using gentle tapping, as needed. Both groups covered similar distances. Exercise sessions occurred 5 days per week from 17:00–20:00 over a total of 5 weeks, while maintaining the original diet throughout the exercise period.

### Animal phenotype

Body weight was measured at a fixed time every week, and random blood glucose (RBG) and fasting blood glucose (FBG) were measured every two weeks. Food intake was monitored but excluded from analysis due to high variability caused by technical challenges (e.g., spillage). Intraperitoneal glucose tolerance test (IPGTT) and insulin tolerance test (IPITT) were performed as prescribed before [32]. Briefly, IPGTT was performed after a 16 h-fasting. Mice were intraperitoneally injected with glucose (2 g/kg), and blood glucose was measured after glucose injection at 0, 10, 20, 30, 60, 90 and 120 min from tail tip via glucometer and strips (ACCU-CHEK, Roche). ITT was performed after a 6 h-fasting. Mice were intraperitoneally injected with 0.5 U/kg insulin, and blood glucose was measured after insulin injection at 0, 15, 30, 60, 90 and 120 min. The area under the curve (AUC) was determined using GraphPad Prism 9.3.0 software.

### Serum biochemical analysis

The blood samples were centrifuged at 3000 r/min for 30 min at 4°C to acquire the serum samples which were then stored at -80°C. Serum samples were diluted four-fold and analyzed with an automated biochemical analyzer (Roche Cobas 702, Germany) to measure serum cholesterol (CHO), high-density lipoprotein (HDL) cholesterol, low-density lipoprotein (LDL) cholesterol, triglycerides (TG), alanine aminotransferase (ALT), aspartate aminotransferase (AST), creatine kinase (CK), lactate dehydrogenase (LDH), and lactate (LACT) using corresponding kits (Roche, Germany) according to the manufacturer’s guidelines. Serum insulin levels were measured using the Mouse Insulin Ultrasensitive ELISA Kit (Catalog number: 90080, Crystal Chem, USA) followed the manufacturer’s guidelines.

### Histology

Fresh liver and adipose tissue were extracted and fixed with 4% paraformaldehyde immediately for more than 24 h. Part of these tissues were dehydrated by gradient concentrations of ethanol and xylene, embedded in the paraffin and processed to 5 μm sections to stained with hematoxylin and eosin (HE) by standard procedure [28]. The other part of these tissues was embedded in OCT compound (Sakura Finetek, USA) for cryostat sectioning. 5 μm sections from the OCT-embedded blocks were stained with oil red O as reported [5]. The images were visualized and captured using an optical microscope (Nikon, Japan). The area of adipocyte and the oil res O staining on liver were analyzed with Image J.

### Primary cell extraction, culture and lipid induction

Primary adipocytes were isolated from paragluteal adipose tissue, interscapular brown adipose tissue of suckling mice. The fat pads were removed, minced, and digested using collagenase I (1.5mg/ml, Solarbio, China) at 37°C for 30 min with shaking. The primary adipocytes were then adequately washed and cultured in Dulbecco’s modified eagle medium (DMEM) containing 20% fetal bovine serum (FBS) and 1% penicillin-streptomycin solution. For primary white adipocytes (PWA), cells were cultured in introduction media I (DMEM plus 10% FBS, 0.5 mmol/l IBMX, 10 μg/ml insulin, 1μmol/l dexamethasone) for 48 hours, then cultured in introduction media II (DMEM plus 10% FBS, 10 μg/ml insulin) for 2–3 days, and then finally switched to the DMEM with 10% FBS for maintenance. The differentiation of primary brown adipocytes (PBA) follows the same procedure above except for the additional addition of 1 nmol/l T3 to the introduction medium I and II.

### RNA extraction, reverse transcription, and real time-qPCR

Total RNA of liver, adipose tissue, muscle (20–40 mg) and primary adipocytes were extracted with the Total RNA Extraction Kit (Axygen, USA). cDNA was prepared from 1000 ng RNA by HiScript III RT SuperMix for qPCR (+gDNA wiper) (Vazyme, China). Real-time qPCR was performed using SYBR Green premixed system (Bio-rad, USA). All procedures followed the manufacturer’s protocols. Gene expression was presented with the ΔΔCT method and normalized to β-actin. Primers are listed in S2 Table in S1 File.

### Statistical analysis

The results were expressed as mean ± SEM. Statistics were analyzed by GraphPad Prism Version 8. For normally distributed data, t-tests were used to compare the means of two groups, and one-way ANOVA with Tukey’s multiple comparison test was used for comparisons between three or more groups; for repeated-measurement data, ANOVA for repeated-measurement data was used.

## Results

### HIIT and MICT alleviate body weight gain and tissue weight gain

After 12 weeks of dietary intervention, HFD-fed mice gained significantly more weight than CD-fed mice. Additionally, both RBG and FBG in HFD-fed mice were significantly higher than those in CD-fed mice (S1A–D Fig in S1 File). Next, some of HFD-fed mice were randomly assigned to undergo 5 weeks of MICT or HIIT.

**Fig 1:**
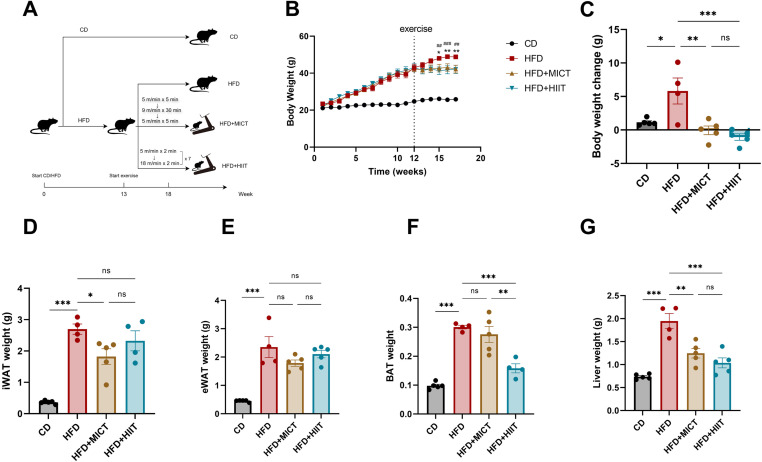
HIIT and MICT alleviate body weight gain and tissue weight gain. (A) Experimental design. (B) Weight gain curve of mice. (C) Body weight change after 5-week exercise. (D-G) Wet weight of iWAT, eWAT, BAT and liver. *p<0.05, **p<0.01, ***p<0.001. n=4-5 per group.

At the end of the 5 weeks of exercise period, the body weights of mice in both MICT and HIIT groups were significantly lower than those in HFD group (p = 0.002), with no significant difference between MICT and HIIT groups (Fig 1B, C). Adipose tissue and liver wet weights in HFD-fed mice were significantly higher than those in CD-fed mice (p < 0.001). MICT significantly reduced inguinal white adipose tissue (iWAT) weight (p = 0.049) while HIIT not. However, both MICT and HIIT did not alter the epididymal white adipose tissue (eWAT) weights (Fig 1D, E). BAT weight was significantly lower in the HIIT group than those in HFD group (p < 0.001), whereas MICT group did not show significant difference (Fig 1F). Liver weight was significantly lower in both MICT and HIIT groups (p = 0.002 vs. p < 0.001, respectively), but there was no significant difference between the groups (Fig 1G). These findings suggested that different types of adipose tissues exhibit varying sensitivities to MICT and HIIT. MICT was more effective in reducing the weight of WAT, HIIT significantly decreases the weight of BAT. And the reduction in liver weight is comparable between MICT and HIIT.

### HIIT is superior to MICT in improving insulin sensitivity with comparable benefits on serum lipids

To determine the effects of MICT and HIIT on glycolipid metabolism, we measured serum lipids and performed the IPGTT and IPITT tests. TG, LDL, HDL, and CHO were significantly elevated in HFD group compared to CD group. Both MICT and HIIT groups demonstrated significant lower levels of LDL and TG compared to HFD group; however, there was no significant difference on CHO and HDL levels. Additionally, no significant differences were observed between the MICT and HIIT groups (Table 1).

**Table 1:** Serum lipids in mice.

	CD	HFD	p value*vs.*CD	MICT	p value *vs.*HFD	HIIT	p value *vs.*HFD	p value MICT *vs.*HIIT
CHO, mg/dl	87.44 ± 6.46	210.60 ± 22.68	<0.001	173.80 ± 9.78	0.300	177.80 ± 15.18	0.394	0.997
HDL, mg/dl	63.84 ± 4.18	130.60 ± 16.14	0.003	117.20 ± 10.98	0.813	135.30 ± 9.43	0.990	0.598
LDL, mg/dl	12.00 ± 1.69	32.00 ± 3.22	<0.001	21.12 ± 1.69	0.042	19.92 ± 3.22	0.022	0.985
TG, mg/dl	81.50 ± 6.70	115.10 ± 9.11	0.021	80.80 ± 2.86	0.025	81.60 ± 8.13	0.029	0.999

**Fig 2:**
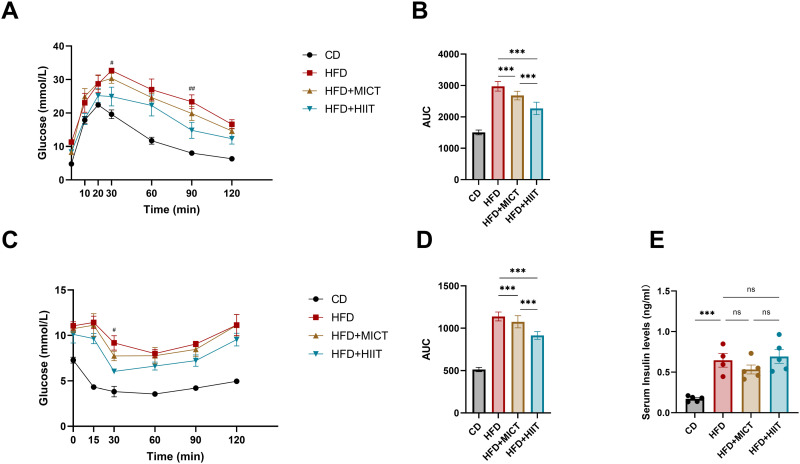
Effect of HIIT and MICT on glucose tolerance, insulin sensitivity and fasting serum insulin levels. (A-B) Intraperitoneal glucose tolerance test and area under curve. (C-D) Intraperitoneal insulin tolerance test and area under curve. (E) Fasting serum insulin levels. ^#^p < 0.05, ^##^p < 0.01 *vs.* HIIT, * p < 0.05, **p < 0.01, ***p < 0.001. n = 4–5 per group.

The IPGTT results showed that the blood glucose levels curves in HFD-fed groups were consistently higher than those in the CD group, with delayed peaks. Both MICT and HIIT groups displayed lower in blood glucose levels compared to the HFD group. Notably, HIIT significantly reduced blood glucose levels compared to HFD group (p = 0.015 at 30min and 0.006 at 90min), with improved delayed peaks. Moreover, the AUC analysis indicated that both MICT and HIIT enhanced overall glucose metabolism, with the HIIT group showing a significantly greater reduction in blood glucose levels (p < 0.001) (Fig 2A, B). Similar trends were observed in the IPITT, where blood glucose levels in HFD-fed groups were significantly elevated compare to CD group, and HIIT significantly reduced blood glucose levels compared to HFD group (p = 0.011 at 30min). The AUC of blood glucose in the HIIT group was significantly lower than that in both MICT and HFD groups (p < 0.001) (Fig 2C, D). Additionally, fasting serum insulin levels were significantly elevated in HFD-fed groups, with no intergroup differences noted (Fig 2E).

Together, these results suggest that both HIIT and MICT can alleviate high-fat-induced hyperlipidemia, glucose intolerance and insulin resistance with HIIT exhibiting superior efficacy on insulin sensitivity. However, neither intervention affected fasting serum insulin levels.

### HIIT is superior to MICT in alleviating high-fat-induced hepatic steatosis with comparable benefits on liver function impairment

**Fig 3:**
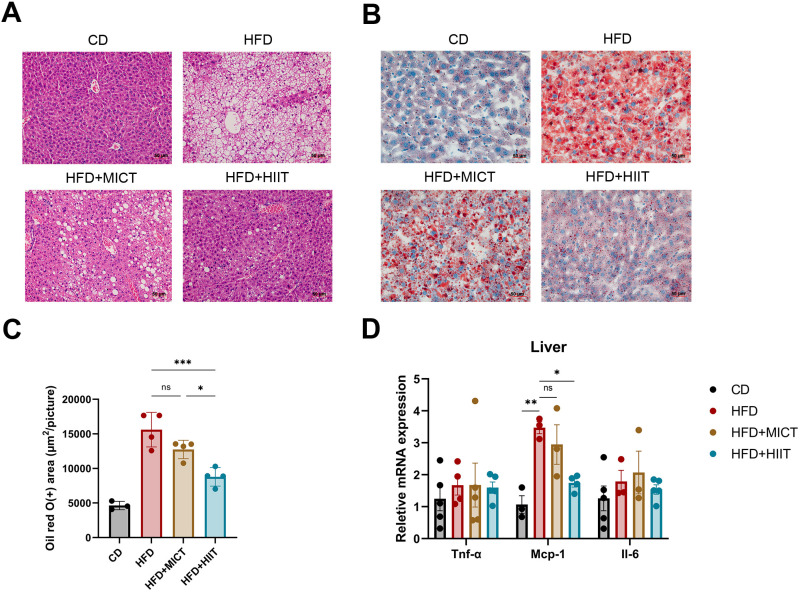
Effects of HIIT and MICT on liver pathology and inflammatory factor gene expression. (A) HE staining of liver (scale bar: 50μm, magnification: 10×). (B) Oil red O staining of liver (scale bar: 50μm, magnification: 10×). (C) Quantitative analysis of oil red O (+) area. (D) mRNA levels of inflammatory factors Tnf-α, Mcp-1, Il-6 in liver. * p < 0.05, **p < 0.01, ***p < 0.001. n = 3–5 per group.

HE staining of liver section from HFD group revealed severe steatosis, cytoarchitectural disruption, fibrotic changes, and inflammatory cells infiltration compared to CD group. Both MICT and HIIT attenuated these lesions, with HIIT demonstrating a more pronounced effect (Fig 3A). Oil Red O staining demonstrated substantial lipid deposition in the livers of HFD group, while both MICT and HIIT groups showed notable reductions in lipid deposition (p = 0.366 and < 0.001, respectively), with HIIT achieving a greater reduction than MICT (p = 0.030) (Fig 3B, C).

The liver function indicators were also measured. Serum ALT, AST CK and LDH were significantly elevated in HFD group, whereas both MICT and HIIT groups demonstrated significant reductions, returning to levels comparable to the CD group, with no differences between MICT and HIIT groups (Table 2). We also examined the mRNA levels of inflammatory factors in the liver, including Mcp-1, Tnf-α and Il-6. Mcp-1 levels were significantly elevated in HFD group (p = 0.004), and HIIT significantly decreased Mcp-1 levels (p = 0.017) while MICT did not alter the Mcp-1 levels (Fig 3D). The mRNA levels of Tnf-α and Il-6 did not show significant difference between groups (Fig 3D).

**Table 2:** Liver function biochemical parameters in mice.

	CD	HFD	p value *vs.*CD	MICT	p value *vs.*HFD	HIIT	p value *vs.*HFD	p value MICT *vs.*HIIT
ALT, U/L	20.40 ± 5.16	194.93 ± 27.27	<0.001	68.60 ± 13.57	<0.001	49.20 ± 13.52	<0.001	0.763
AST, U/L	117.20 ± 15.59	366.10 ± 28.94	<0.001	155. 60 ± 9.48	<0.001	136.10 ± 5.64	<0.001	0.832
CK, U/L	1548.00 ± 303.61	4079.00 ± 995.85	0.016	1599.00 ± 425.20	0.018	1344.00 ± 104.90	0.009	0.982
LDH, U/L	549.60 ± 56.15	1673.00 ± 134.98	<0.001	720.00 ± 84.19	<0.001	518.40 ± 58.06	<0.001	0.357
LACT, mg/dl	101.27 ± 12.10	189.03 ± 10.59	0.121	145.00 ± 40.79	0.632	92.10 ± 17.69	0.099	0.490

Together, these results indicated that both MICT and HIIT can reverse the liver function impairment, while HIIT provided superior mitigation of hepatic steatosis. Additionally, HIIT reduced the mRNA levels of Mcp-1 in liver, suggesting that HIIT may have the potential to reduce liver inflammation markers which needs further verification.

### Divergent effects of MICT and HIIT on high-fat-induced pathological remodeling in iWAT and BAT

**Fig 4:**
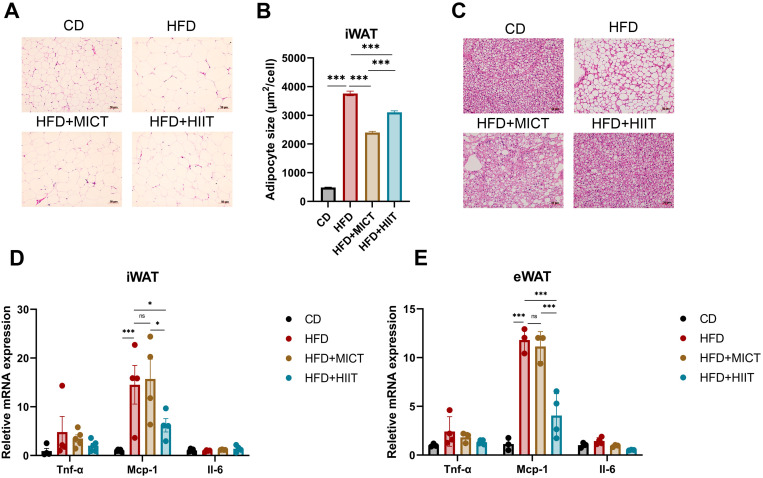
Effects of HIIT and MICT on adipose tissues pathology and inflammatory factor gene expression. (A-B) HE staining of iWAT and quantitative analysis of adipocyte size (scale bar: 50μm, magnification: 10×). (C) HE staining of BAT (scale bar: 50μm, magnification: 10×). (D-E). mRNA levels of inflammatory factors Tnf-α, Mcp-1 and Il-6 in iWAT and eWAT. * p < 0.05, **p < 0.01, ***p < 0.001. n = 3–5 per group.

We performed HE staining of iWAT and BAT. In HFD-fed groups, adipocyte size in iWAT showed a significant increase; however, the size in both MICT and HIIT groups was significantly lower compared to HFD group (p < 0.001). Quantitative analysis revealed that MICT had a more pronounced attenuating effect than HIIT (p < 0.001) (Fig 4A, B). Similarly, brown adipocyte size in HFD-fed groups also increased, accompanied by a decrease in the number of cells per unit area, with large and homogeneous LD, indicating characteristics of WAT. These lesions were mitigated by both MICT and HIIT, with a more pronounced effect in HIIT group (Fig 4C).

Next, we measured the mRNA levels of the inflammatory factors Mcp-1, Tnf-α, and Il-6 in iWAT, eWAT and BAT. Mcp-1 was significantly upregulated in both iWAT and eWAT of HFD group (p < 0.001), and HIIT significantly reduced this high-fat-induced upregulation in both iWAT (p = 0.016) and eWAT (p < 0.001). However, no significant difference was observed between HFD and MICT group. (Fig 4D, E).

These results indicated that both MICT and HIIT can alleviate high-fat-induced adipocyte hypertrophy in WAT and BAT, with MICT being more effective in WAT and HIIT being more effective in BAT. As in the liver, only HIIT significantly ameliorated the elevation of Mcp-1 in mRNA levels in WAT, indicating the potential of HIIT in reducing inflammation markers in WAT which needs further verification.

### Effects of HIIT and MICT on lipid metabolism in various tissues

**Fig 5:**
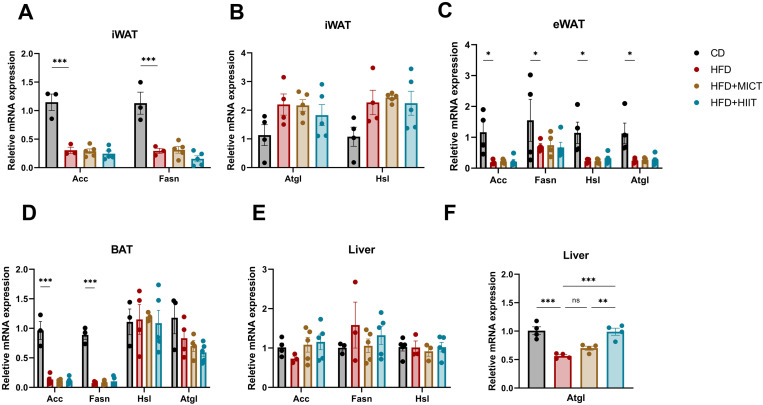
Effects of HIIT and MICT on lipid synthesis and catabolism. (A-B) mRNA levels of Acc, Fasn, Atgl and Hsl in iWAT. (C-D) mRNA levels of Acc, Fasn, Atgl and Hsl in eWAT and BAT. (E-F) mRNA levels of Acc, Fasn, Atgl and Hsl in liver. * p < 0.05, **p < 0.01, ***p < 0.001. n = 3–5 per group.

We examined the mRNA levels of key lipogenesis enzymes, including Acc and Fasn, as well as key lipolysis enzymes Hsl and Atgl. In both iWAT and eWAT, Acc and Fasn were significantly downregulated in HFD group compared to CD group (p < 0.001). Additionally, Atgl and Hsl were significantly downregulated in eWAT (p = 0.034 and p = 0.027, respectively), and no significant changes were observed in iWAT. However, neither MICT nor HIIT altered the expression of these genes compared to HFD group (Fig 5A–C). In BAT, Acc and Fasn were also significantly downregulated in HFD group (p < 0.001), and no altered expression was observed among them. Hsl and Atgl did not significantly differ between HFD and CD groups; however, HIIT group exhibited a trend toward downregulation of Atgl expression (p = 0.059) (Fig 5D).

Notably, while Acc, Fasn, and Hsl did not show significant differences among the four groups in the liver, Atgl was significantly downregulated (p < 0.001), and HIIT significantly reversed this downregulation (p < 0.001) (Fig 5E, F). Taken together, these findings suggest that 5 weeks of exercise does not significantly affect the mRNA levels of lipogenesis and lipolysis genes in adipose tissues. However, HIIT significantly restored the high-fat-induced downregulation of Atgl, suggesting that HIIT may enhancing the lipolysis in liver, which could be a potential mechanism of improvement of hepatic steatosis and lipid deposition by HIIT.

### Impact of HIIT and MICT on Cidea, Cidec

We investigated the effects of exercise on Cidea, Cidec and related transcription factors in adipose tissues and liver.

**Fig 6:**
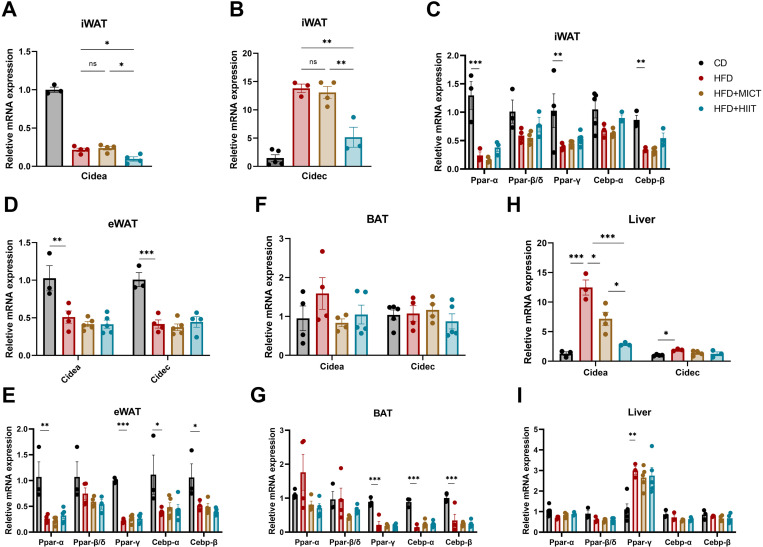
Cidea and Cidec expressions profiles in adipose tissues and liver. mRNA levels of Cidea and Cidec in iWAT (A-B), eWAT (D), BAT (F) and liver (H); mRNA levels of Ppar-α, Ppar-β/δ, Ppar-γ, Cebp-α and Cebp-β in iWAT (C), eWAT (E), BAT (G) and liver (I). * p < 0.05, **p < 0.01, ***p < 0.001. n = 3–5 per group.

In iWAT, Cidea was significantly downregulated, while Cidec was significantly upregulated in HFD groups (p < 0.001). HIIT significantly attenuated the high-fat-induced upregulation of Cidec and further downregulated Cidea (p = 0.001 and 0.044, respectively), whereas MICT did not significantly affect either Cidea or Cidec (Fig 6A, B). Neither MICT nor HIIT had a significant effect on the related transcription factors (Fig 6C). In eWAT, the HFD group exhibited significant downregulation of Cidea and Cidec (p = 0.005 and < 0.001, respectively), with a consistent trend observed on the transcription factor. However, neither MICT nor HIIT groups showed altered expression of Cidea, Cidec or related transcription factors compared to HFD group (Fig 6D, E). No differences were observed among four groups in Cidea, Cidec levels in BAT, but Ppar-γ, Cebp-α and Cebp-β were downregulated in all HFD-fed groups (p < 0.001), which may not relate to Cidea and Cidec. (Fig 6F, G). In the liver, Cidea was significantly upregulated in the HFD group (p < 0.001), and both MICT and HIIT attenuated the upregulation of Cidea (p = 0.012 and < 0.001, respectively), with HIIT showing a more significant effect (p = 0.036). Cidec exhibited a similar tendency without significant differences (Fig 6H). Ppar-γ was significantly upregulated in HFD group, and both MICT and HIIT showed a trend toward reducing the upregulation, consistent with the trends observed for Cidea and Cidec (Fig 6I).

Taken together, these suggest that high-fat induce different expression patterns of Cidea and Cidec in various adipose tissues and liver. Furthermore, HIIT could downregulate Cidea and Cidec in iWAT and liver, while MICT only alleviated the upregulation of Cidea in liver.

### Il-6 downregulates Cidec and upregulate Atgl in white adipocytes

**Fig 7:**
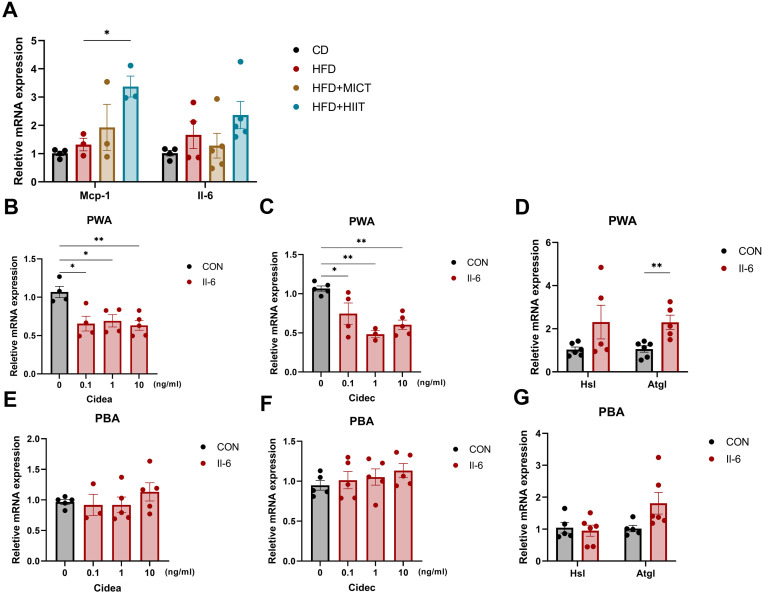
Cidea, Cidec and Atgl expression of primary adipocyte with addition of Il-6. (A) mRNA levels of Mcp-1 and Il-6 in skeletal muscle. (B-C) mRNA levels of Cidea and Cidec in primary white adipocyte with addition of different concentration of Il-6. (D-G) mRNA levels of Atgl in primary white adipocyte and brown adipocyte. (E-F) mRNA levels of Cidea and Cidec in primary brown adipocyte with addition of different concentration of Il-6. * p < 0.05, **p < 0.01, ***p < 0.001. n = 4–6 per group.

Skeletal muscle is a key locomotor organ, so we examined the expression of genes related to glucose and lipid metabolism in the femoral quadriceps; however, no significant difference in the mRNA levels of those genes (S2A, B Fig in S1 File). Given that skeletal muscle has been recognized as a secretory organ, we also assessed the mRNA levels of Mcp-1 and Il-6 in skeletal muscle. The results showed that MICT did not significantly alter the expression of Mcp-1 and Il-6, whereas HIIT significantly upregulated Mcp-1 (p = 0.038) compared to HFD group (Fig 7A).

We sought to understand how exercise influenced Cidea and Cidec expression. Previous studies have shown that exercise can lead to substantial secretion of Il-6 [33]. Additionally, Il-6 has been shown to promote lipolysis, although the specific mechanism remains unclear [34,35]. Tnf-α, which also promote lipolysis, has been reported to phosphorylate PPARγ via the MEK/ERK pathway, enhancing lipolysis in adipocytes and reducing Cidec expression [36]. Therefore, we hypothesized that Il-6 might act on adipose tissue to reduce Cidea and Cidec expression and promote adipocyte lipolysis.

We extracted primary adipocytes from suckling mice and induced them to differentiate into PWA and PBA (S2C, D Fig in S1 File). We added different concentrations of Il-6 to the cell culture medium, and results showed that Cidea and Cidec in PWA were significantly downregulated after the addition of Il-6, with no change observed with varying drug concentration (Fig 7B, C). Furthermore, Atgl expression was also significantly upregulated at the mRNA level (p = 0.005), indicating increased lipolysis (Fig 7D). In contrast to PWA, expression of Cidea and Cidec in PBA was not significantly altered (p = 0.069), while Atgl expression tended to increase, although these changes were not statistically significant (Fig 7E–G).

## Discussion

### Exercise improves obesity and related metabolic disorders, and HIIT is superior to MICT in improving insulin sensitivity, and promoting lipolysis

In the present study, consistent with previous studies [12,37,38], we confirmed the significant benefits of MICT and HIIT in obesity-related metabolic dysfunction. Our findings indicate that HIIT demonstrated more pronounced improvements in insulin sensitivity, hepatic steatosis and BAT structure, whereas MICT is more effective in reducing WAT weight and improving WAT structure. HIIT also showed potential to reduce inflammation markers in targeted tissue, however, the association of HIIT and inflammation state need further investigation through histological assessment of immune cell infiltration and other direct measurement in the future study. Previous studies have reported mixed results regarding the effects of MICT and HIIT on body weight and fat mass, with some studies indicating HIIT is superior for fat loss [8,9], while others reported comparable or negligible effects [10–12], and there is study reported superior results with MICT [39]. Our data revealed divergent effects of MICT and HIIT on adipose tissue-specific fat loss. Notably, MICT significantly decreased iWAT mass and adipocyte size, whereas HIIT primarily reduced BAT mass and adipocyte size. This discrepancy may be due to different physiological mechanisms: MICT likely induces a sustained caloric deficit, making WAT - a primary energy reservoir - more responsive to chronic negative energy balance and preferentially mobilised for fuel supply. In contrast, HIIT induces acute sympathetic activation with elevated catecholamine levels, which predominantly activates BAT due to its dense sympathetic innervation and increased adrenergic sensitivity [40,41]. BAT as a thermogenically active organ, is critical for countering metabolic disorders. In our study, it exhibited reduced mass following HIIT intervention, yet HIIT correlated with metabolic improvements. This contradiction may be explained by two non-exclusive mechanisms: 1) HIIT-induced benefits in non-adipose organs (e.g., glucose uptake and liver), and 2) functional enhancement of BAT despite mass reduction. Notably, advances in BAT measurements have challenged the historical view that BAT mass directly reflects its thermogenic capacity [42,43]. Emerging evidence from diet-induced obese rodent models demonstrates that obesity-driven BAT expansion often coincides with adipocyte whitening and impaired thermogenesis due to chronic energy surplus. Conversely, HIIT may alleviate lipid overload in BAT by acutely elevating energy demands, thereby restoring mitochondrial uncoupling efficiency and suppressing whitening pathways. Thus, BAT mass reduction under HIIT likely represents a functional optimization rather than degenerative loss.

Previous studies have demonstrated that both MICT and HIIT upregulate the expression of adipogenic genes Acc and Fasn [44,45]. However, our study observed no significant alterations in Acc or Fasn expression following either intervention. This discrepancy could stem from methodological variations: compared to prior protocols, our exercise duration was shorter (5 vs. 8 weeks) and high-fat diet (HFD) exposure longer (17 vs. 12–15 weeks). Sustained HFD likely induced chronic exogenous lipid overload, triggering feedback inhibition of endogenous lipogenesis in adipose tissue. The current exercise protocol’s intensity and duration may be insufficient to counteract this suppression under persistent hyperlipidemic conditions. In contrast, hepatic Acc and Fasn expression remained unaltered in HFD-fed mice, likely attributable to hyperglycemia-driven activation of de novo lipogenesis pathways that counterbalanced exogenous lipid-induced suppression. Still, neither MICT nor HIIT significantly altered Acc or Fasn expression in liver. These suggest that, at least in the absence of dietary intervention, the fat-loss effects of MICT and HIIT in this study were not mediated through changes in lipogenesis.

The lipolytic key enzyme genes Hsl and Atgl in HFD-fed mice showed lower expression levels in eWAT and higher expression levels in iWAT compared to CD-fed mice, suggesting that a difference in lipolytic potential between iWAT and eWAT. MICT and HIIT did not significantly change their transcript levels, while previous studies have shown enhanced lipolytic ability, suggesting that other mechanisms, such as post-translational modifications (PTM), may regulate their lipolytic capacity [46]. Additionally, our sampling was conducted one day post-exercise which may obscure potential alterations since exercise-induced genes expression alterations could be time-limited. Consistent with previous study [47], exercise markedly enhanced hepatic Atgl expression, and we observed HIIT elicited a more pronounced elevation in Atgl expression. Conversely, it has also been demonstrated that Atgl is elevated in high-fat induced obese mice, and exercise effectively reversed this upregulation [30]. This may be attributed to differences in the exercise protocols where some study showed that HIIT led to even lower body weight in HFD group than CD group, which is not considered a healthy outcome of the exercise intervention. Consequently, alterations in the transcription factors levels may be a compensatory response. In conclusion, our findings suggest that the liver is particularly responsive to HIIT in promoting lipolysis, while no alterations in lipid metabolism-related transcription factors were observed [45].

### The effects of high fat on Cidea and Cidec are tissue-specific, and exercise can alter their expression in some tissues

We are the first to comprehensively discuss the effects of high fat and exercise on the Cidea/Cidec expression profiles of specific types of adipose tissue. The key findings reveal depot-specific responses: in iWAT, HFD upregulated Cidec while downregulating Cidea, whereas HIIT significantly downregulated both isoforms. The reduction due to HIIT may be attributed to the export of lipids from adipose tissue for catabolism, thereby supplying substantial energy for high-intensity exercise. The HFD-mediated downregulation of Cidea may represent a compensatory mechanism, consistent with previous reports [48]. In contrast with previous findings [48], we observed reduced expression of Cidea and Cidec in eWAT induced by HFD, possibly due to lower lipid absorption and storage capacity compared to iWAT. Cidea and Cidec were downregulated in an attempt to resist excessive lipid accumulation. Neither MICT nor HIIT altered Cidea and Cidec expression, and this because be that the impact of short-term exercise on eWAT may manifest later than that on iWAT. Nevertheless, further investigation is necessary to elucidate mechanisms behind these observed differences. In BAT, neither the HFD nor exercise resulted in a statistically significant difference in the expression of Cidea and Cidec. However, Cidea proteins have been reported to be significantly elevated following HFD [49]. The difference may be attributed to PTM, as Cidea proteins could be regulated by ubiquitin-mediated proteasomal degradation pathways [50]. Cidea in BAT were reported to negatively regulate energy expenditure by promoting degradation of the AMPK complex [49], and this may explain the observed trend towards lower Cidea after exercise. This also suggests that Cidea plays other roles in BAT besides promoting LD fusion.

The Cidea/Cidec expression profile of in liver is consistent with previous study. Furthermore, our findings showed that HIIT demonstrated greater resilience to the HFD-induced upregulation of Cidea compared to MICT. Cidea and Cidec proteins can accelerate the fusion of LD in hepatocytes and adipocytes and attenuate mitochondrial fatty acid β-oxidation (FAO) and peroxisomes [51]. Therefore, regulation of Cidea and Cidec may be associated with HIIT-induced alleviation of hepatic steatosis and liver function, but this requires further validation.

### Il-6 mediates the altered expression of Cidea and Cidec in adipocyte and may be associated with regulation of lipolysis

Emerging evidence demonstrates that exercise-induced skeletal muscle secretion of Il-6 exerts systemic metabolic benefits across multiple tissues [33,52]. In heart, Il-6 can protect cardiac mitochondrial function, mitigate oxidative stress and reducing pericardial adipose deposition [53–55]; In adipose tissue, Il-6 enhances visceral fat loss and lipolysis [35]; And Il-6 can also improves glucose homeostasis via glucose transporter type 4-mediated glucose uptake [56] and gastrointestinal glucagon-like peptide-1-dependent insulin secretion [57]. These pleiotropic effects position Il-6 as a critical mediator of inter-tissue metabolic crosstalk. Our findings extend this paradigm by revealing Il-6-associated downregulation of Cidec and upregulation of Atgl in PWA. This inverse relationship suggests a potential regulatory role for Il-6 in modulating white adipocyte lipolytic capacity through Cidec suppression. Given that Cidec promotes lipid droplet fusion and inhibits lipolysis [51], its exercise-mediated reduction may facilitate lipid mobilization, aligning with Il-6’s established role in enhancing fatty acid oxidation.

The present study has several limitations. Firstly, the observed findings were derived exclusively from male mice, which limits insights into potential sex-specific responses and the generalizability of our results to female biological systems. Future investigations need include female mice to address this critical gap. Secondly, the sample size in this study was determined based on preliminary experimental feasibility and empirical observations from our team’s previous work. The small sample size and absence of sample size calculation constitutes a methodological limitation. Future studies will perform sample size calculations to strengthen the statistical robustness. Thirdly, there is a lack of evidence regarding altered circulating levels of exerkines Il-6 before and after the exercise intervention, which would confirm its endocrine role. Fourthly, there is a lack of hepatocyte-related in vitro experiments to further support and explore the mechanism behind the altered expression of Cidea and Cidec. Lastly, while in vitro experiments on primary adipocytes provided initial insights, a deeper investigation into the specific molecular mechanisms is warranted.

## Conclusion

In conclusion, our findings revealed that both exercises modalities effectively alleviated HFD-induced weight gain, dyslipidemia, impaired hepatic function. Notably, HIIT exhibited superior efficacy in improving insulin sensitivity, hepatic steatosis and BAT remodeling, whereas MICT showed greater potency in WAT remodeling. Mechanistically, the HIIT-specific downregulation of Cidea/Cidec in WAT and liver suggests their critical role in mediating exercise-induced metabolic adaptations. Il-6 may serve as a potential critical factor mediating the relationship between exercise and the regulation of Cidea.

## Supporting information

S1 File Figures and tables. (DOCX)

S2 File Raw data for present study. (XLSX)

S3 File Original histological pictures involved. (PDF)
