# Supplementary material for: Comparative effect of high intensity interval training and moderate intensity continuous training on metabolic improvements and regulation of Cidea and Cidec in obese C57BL/6 mice
Source: PLoS One. 2025 Apr 30;20(4):e0322634. doi: 10.1371/journal.pone.0322634 (PMC12043136; doi:10.1371/journal.pone.0322634)
Supplement: S1 File — (DOCX) [file pone.0322634.s001.docx]

| **S1 Table. Nutritions and Composition in diet** | | | | |
| --- | --- | --- | --- | --- |
| Nutritions | XTHF60 | | XTCON50J | |
|  | mass% | energy% | mass% | energy% |
| Protein | 26.23 | 20.12 | 19.20 | 20.10 |
| Carbohydrates | 25.56 | 19.60 | 67.30 | 69.90 |
| Fats | 34.89 | 60.20 | 4.30 | 10.00 |
| Total |  | 100.00 |  | 100.00 |
| Kcal/g | 5.22 |  | 3.85 |  |
| Composition | mass (g) | Energy (Kcal) | mass (g) | energy (Kcal) |
| Casein | 200.00 | 800.00 | 200.00 | 800.00 |
| L-Cystine | 3.00 | 12.00 | 3.00 | 12.00 |
| Corn Starch | 0.00 | 0.00 | 506.20 | 2024.80 |
| Maltodextrin | 125.00 | 500.00 | 125.00 | 500.00 |
| Sucrose | 72.80 | 291.20 | 72.80 | 291.20 |
| Cellulose | 50.00 | 0.00 | 50.00 | 0.00 |
| Soybean oil | 25.00 | 225.00 | 25.00 | 225.00 |
| Lard | 245.00 | 2205.00 | 20.00 | 2205.00 |
| Mineral Premix S10026B | 50.00 | 0.00 | 50.00 | 0.00 |
| Vitamin Premix V10001C | 1.00 | 4.00 | 1.00 | 4.00 |
| Hydrocholine Bitartrate | 2.00 | 0.00 | 2.00 | 0.00 |
| FD&C Red Dye #40 | 0.00 | 0.00 | 0.04 | 0.00 |
| FD&C Yellow Dye #5 | 0.00 | 0.00 | 0.00 | 0.00 |
| FD&C Blue Dye #1 | 0.05 | 0.00 | 0.01 | 0.00 |
| Total | 773.85 | 4037.20 | 1055.1 | 4037.0 |

**S2 Table. RT-qPCR primers sequences**

| Primer | Forward 5’-3’ | Reverse 5’-3’ |
| --- | --- | --- |
| *Hprt* | TCAGTCAACGGGGGACATAAA | GGGGCTGTACTGCTTAACCAG |
| *Act-β* | ACCAGTCCGCCTAGAAGCAC | CGTTGACATCCGTAAAGACC |
| *Il-6* | TGATGCACTTGCAGAAAACA | ACCAGAGGAAATTTTCAATAGGC |
| *Tnf-α* | GGCGGTGCCTATGTCTCA | AGGGTCTGGGCCATAGAA |
| *Mcp-1* | GCTGGAGAGCTACAAGAGGATC | GTCAACTTCACATTCAAAGGTGC |
| *Atgl* | GGTCCTCTGCATCCCTCCTT | AGACATTGGCCTGGATGAGC |
| *Hsl* | GCTGGAGGAGTGTTTTTTTGC | AGTTGAACCAAGCAGGTCACA |
| *Ucp1* | AACTGTACAGCGGTCTGCCT | TAAGCCGGCTGAGATCTTGT |
| *Cpt1a* | CTCCGCCTGAGCCATGAAG | CACCAGTGATGATGCCATTCT |
| *Acc* | CCAGCAGATTGCCAACATC | ACTTCGGTACCTCTGCACCA |
| *Fasn* | GGAGGTGGTGATAGCCGGTAT | TGGGTAATCCATAGAGCCCAG |
| *Ppar-α* | AGAGCCCCATCTGTCCTCTC | ACTGGTAGTCTGCAAAACCAAA |
| *Ppar-β/δ* | GCATGTCGCACAACGCTATC | CGATGTCGTGGATGACAAAGG |
| *Ppar-γ* | CCGTAGAAGCCGTGCAAGAG | GGAGGCCAGCATCGTGTAGA |
| *Cebp-α* | CAAGAACAGCAACGAGTACCG | GTCACTCGTCAACTCCAGCAC |
| *Cebp-β* | ACGACTTCCTCTCCGACCTCT | CGAGGCTCACGTAACCGTAGT |
| *Cidea* | TGCTCTTCTGTATCGCCCAGT | GCCGTGTTAAGGAATCTGCTG |
| *Cidec* | TGTCGTGTTAGCACCGCAG | TTGCGCTGTTCTGATGGGG |
| *Prdm16* | CCCCACATTCCGCTGTGAT | CTCGCAATCCTTGCACTCA |
| *Dio2* | GTGGCTGACTTCCTGTTGGT | GCACACACGTTCAAAGGCTA |
| *Glut4* | GTGACTGGAACACTGGTCCTA | CCAGCCACGTTGCATTGTAG |
| *Pdk4* | AGGGAGGTCGAGCTGTTCTC | GGAGTGTTCACTAAGCGGTCA |
| *Irs1* | TCTACACCCGAGACGAACACT | TGGGCCTTTGCCCGATTATG |
| *Cs* | GGACAATTTTCCAACCAATCTGC | TCGGTTCATTCCCTCTGCATA |


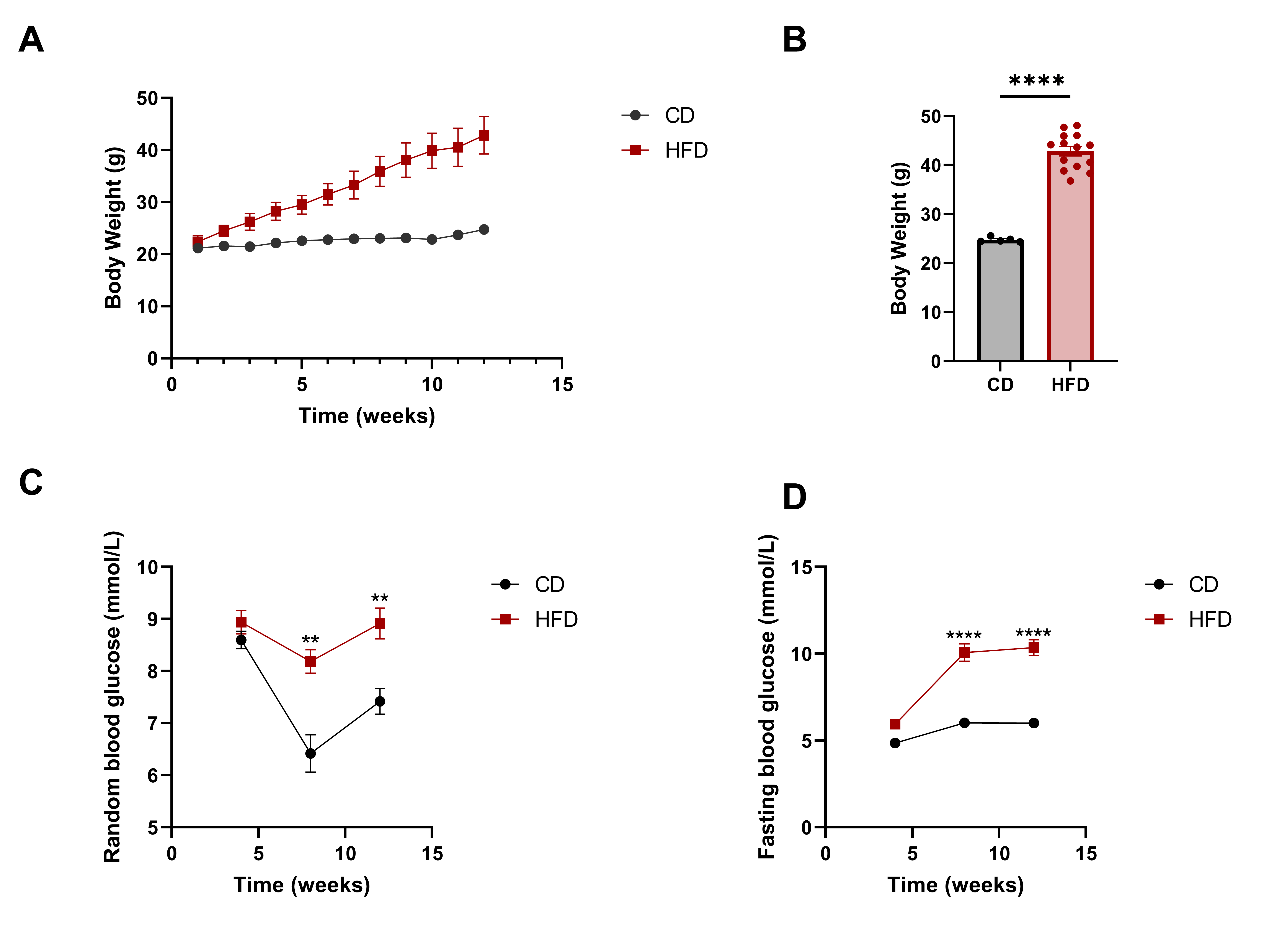


**S1 Fig. Body weight and blood glucose levels after diet intervention**

**(A)** growth curve of body weight. **(B)** Mean body weight of mice after 12 weeks HFD or CD. **(C)** Random blood glucose of mice**. (D)** Fasting blood glucose of mice. ***** p<0.05, **p<0.01, ***p<0.001, **** p<0.0001. n=5 in CD group and n=15 in HFD group.


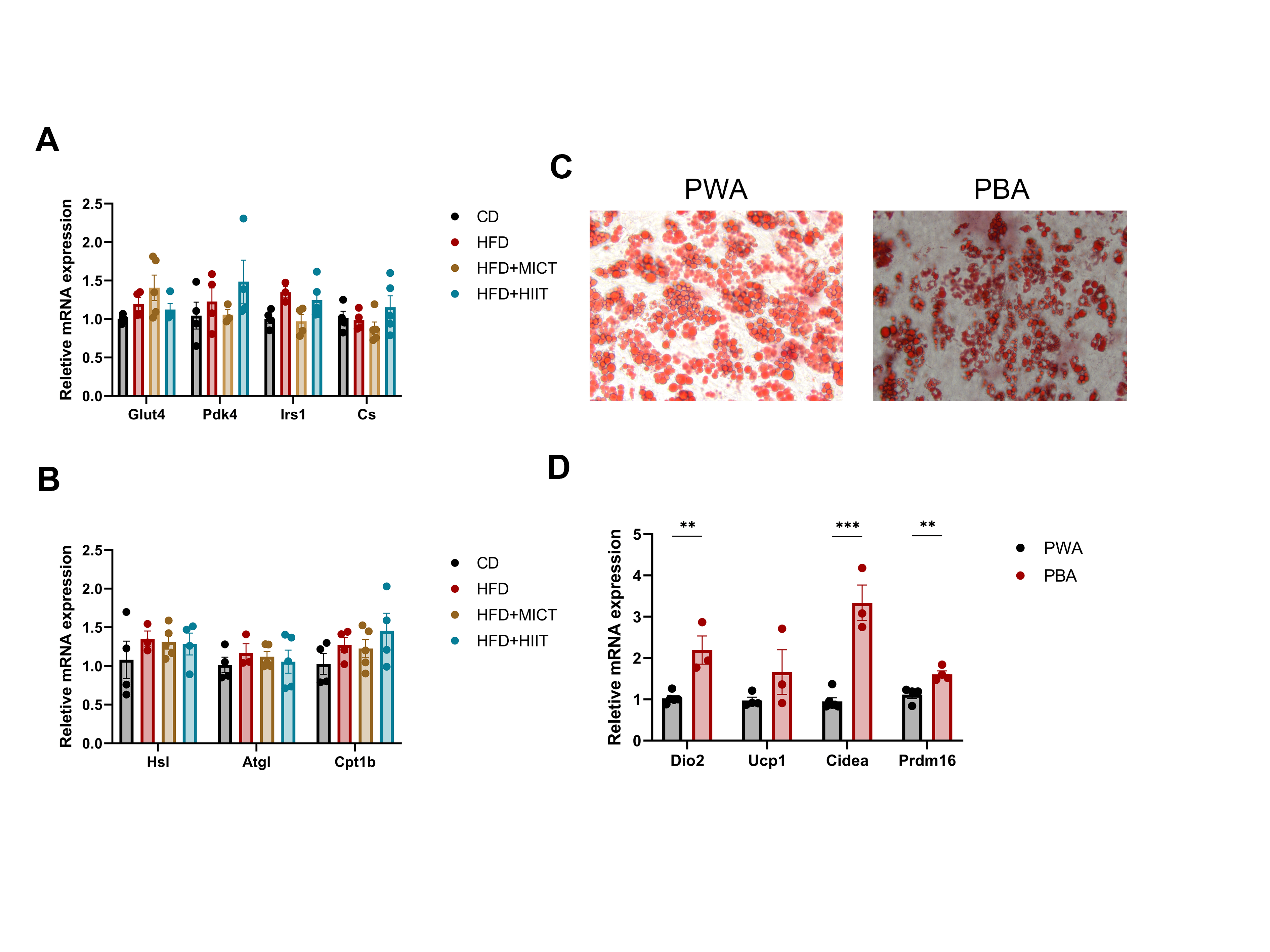


**S2 Fig. Glycolipid metabolism in skeletal muscle and induction of mature primary adipocytes**

**(A-B)** mRNA expression of glucose metabolism genes Glut4, Pdk4, Irs1, Cs and lipid metabolism genes Hsl, Atgl and Cpt1b. **(C)** Oil Red O staining of PWA and PBA**. (D)** mRNA expression of Dio2, Ucp-1, Cidea and Prdm16. ***** p<0.05, **p<0.01, ***p<0.001. n=3-5 per group.
