# Supplementary material for: Comparative effect of high intensity interval training and moderate intensity continuous training on metabolic improvements and regulation of Cidea and Cidec in obese C57BL/6 mice
Source: PLoS One. 2025 Apr 30;20(4):e0322634. doi: 10.1371/journal.pone.0322634 (PMC12043136; doi:10.1371/journal.pone.0322634)
Supplement: S3 File — (PDF) [file pone.0322634.s003.pdf]

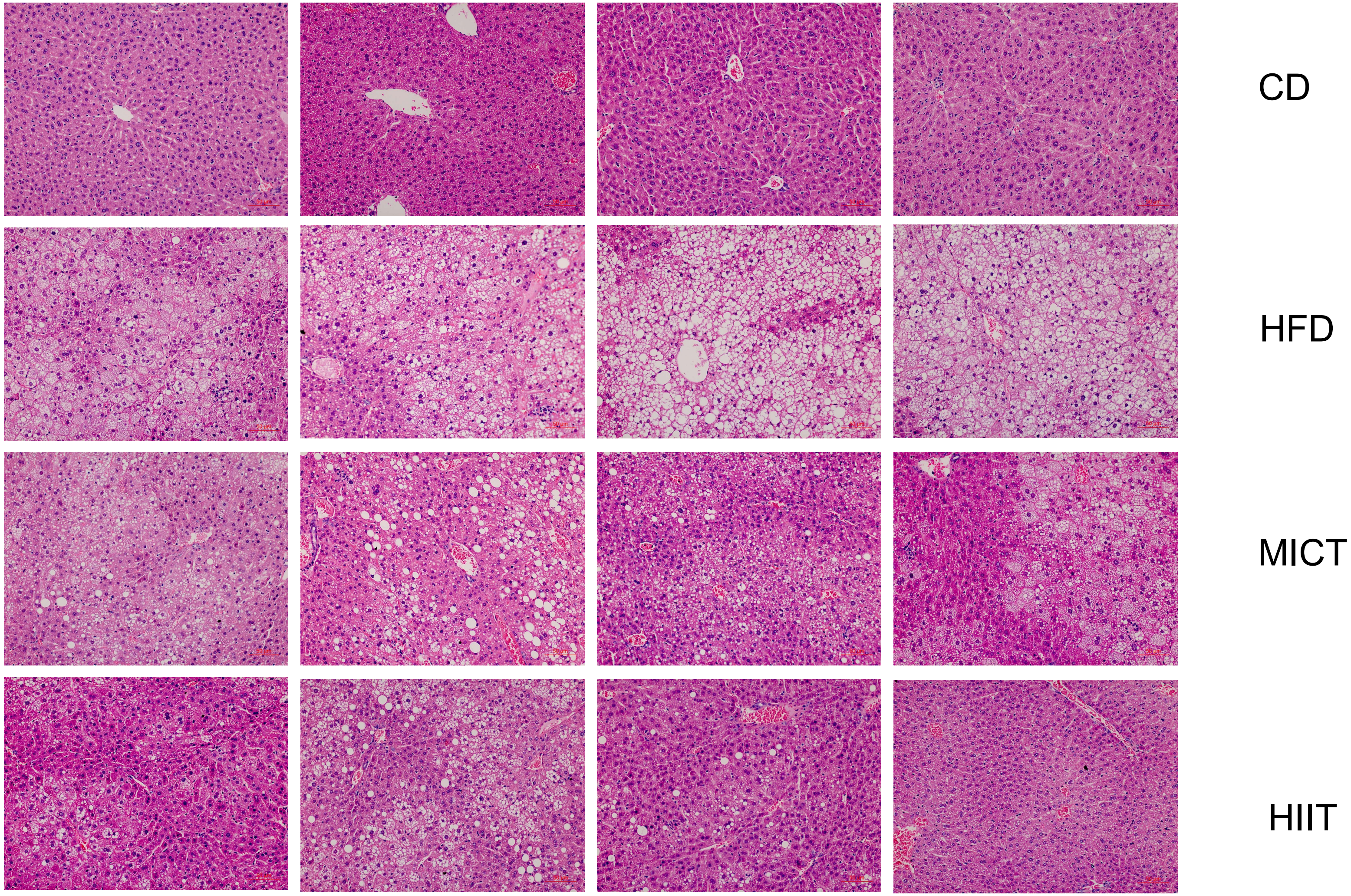

H&E staining (Liver)

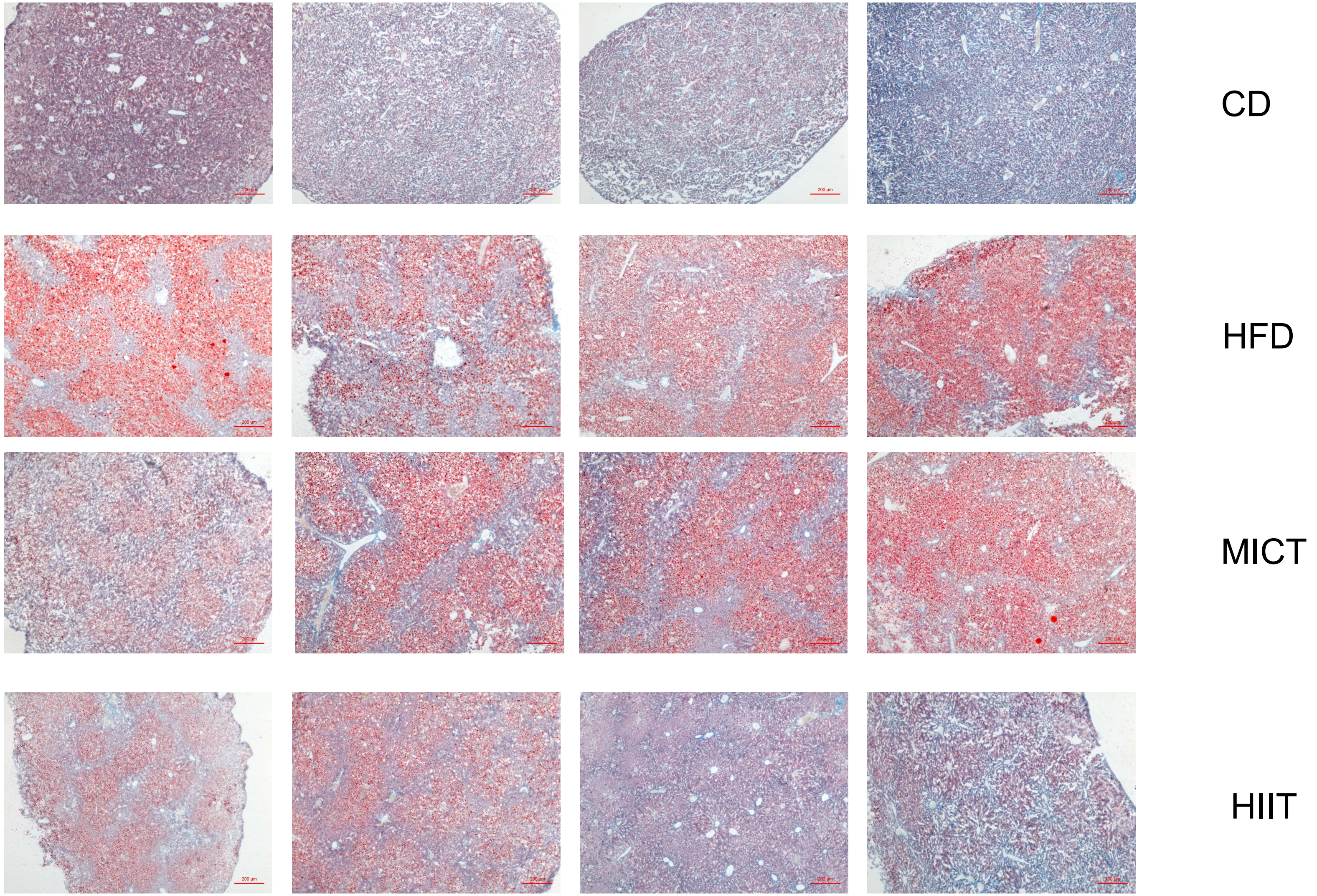

Oil Red staining (Liver)

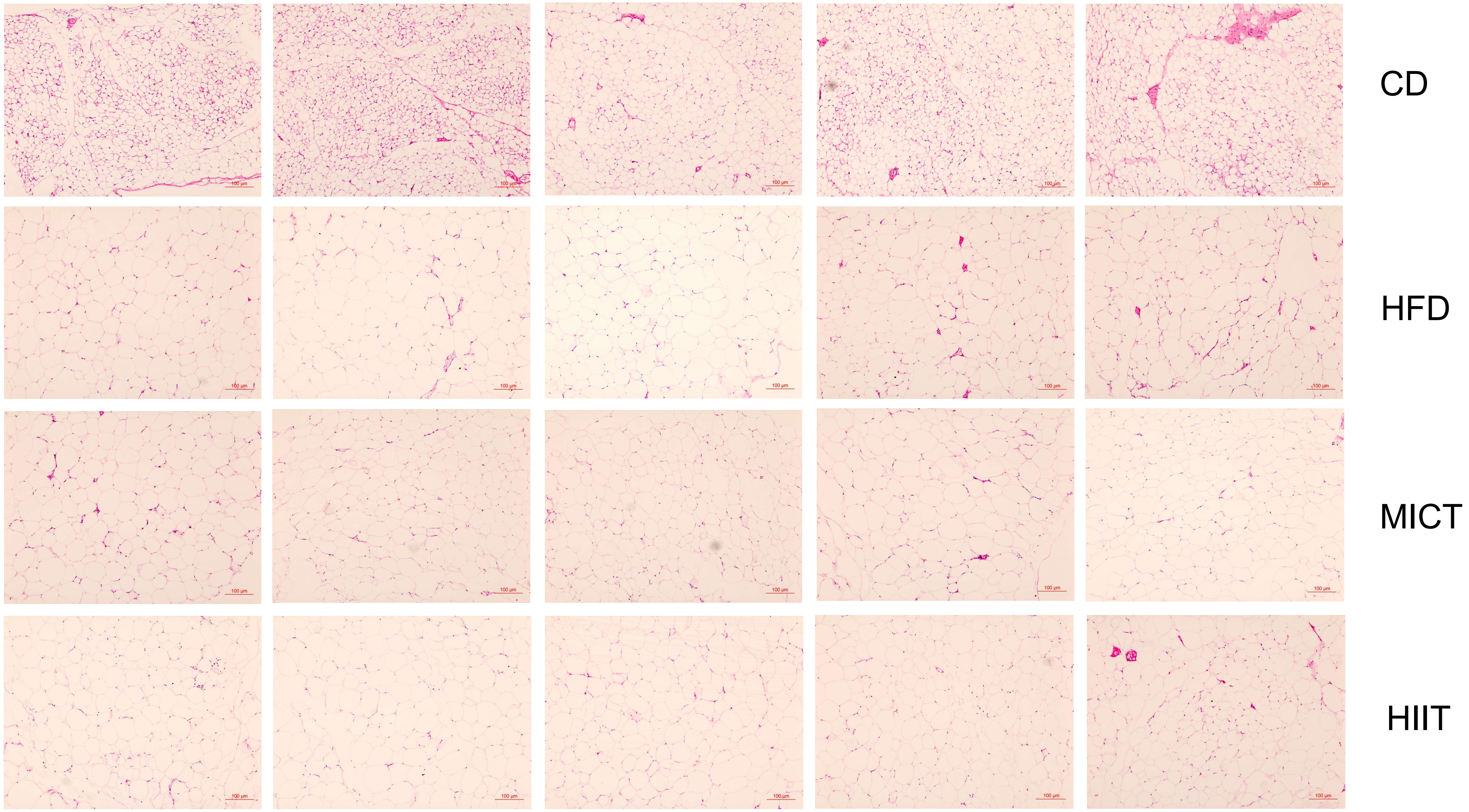

H&E staining (iWAT)

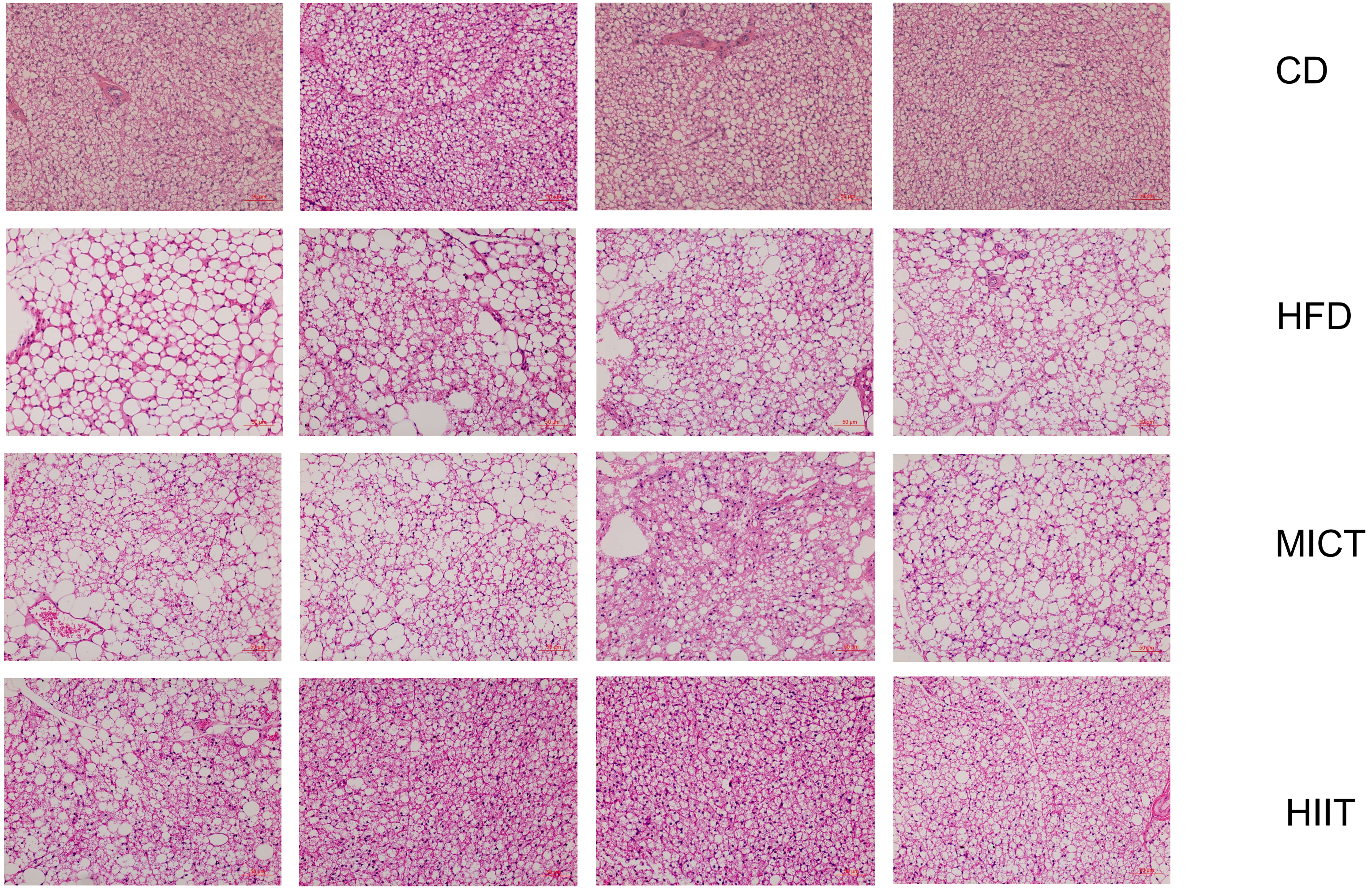

H&E staining (BAT)
